# Problematic social media use and psychological symptoms in adolescents

**DOI:** 10.1007/s00127-024-02657-7

**Published:** 2024-04-07

**Authors:** Ramin Mojtabai

**Affiliations:** Department of Psychiatry and Behavioral Sciences, Tulane University, 1440 Canal Street, Suite 1000, New Orleans, LA 70112 USA

**Keywords:** Social media, Problematic use of social media, Social media addiction, Psychological symptoms, Global mental health

## Abstract

**Purpose:**

This study examined time trends in significant child and adolescent psychological symptoms and explored the association of frequent and problematic social media use with these symptoms.

**Methods:**

Time trends in psychological symptoms were assessed using data from five waves of the international survey of Health Behavior in School-aged Children (HBSC), conducted between 2001 and 2018 (N = 1,036,869). The associations of frequent and problematic social media use with significant psychological symptoms were assessed by hierarchical multinomial logistic regression using data from 2001–2002 and the 2017–2018 survey waves. The direction of effect between social media use variables and psychological symptoms was explored using Linear Non-Gaussian Acyclic Models (LiNGAM).

**Results:**

Prevalence of more severe psychological symptoms increased from 6.7% in 2001–2002 to 10.4% in the 2017–2018 survey waves. The increase was especially large among 15-year old and older girls: from 10.9 to 19.1%. The higher prevalence of more severe psychological symptoms in 2017–2018 compared with 2001–2002 was eliminated after adjusting the model for problematic social media use. LiNGAM analysis supported the direction of effect going from social media use and problematic social media use to psychological symptoms.

**Conclusions:**

The findings suggest that frequent and problematic use of social media contribute to the increasing trend of psychological symptoms in adolescents in recent years.

**Supplementary Information:**

The online version contains supplementary material available at 10.1007/s00127-024-02657-7.

## Introduction

There is growing evidence that the prevalence of child and adolescent psychological symptoms including depressed mood, anxiety, negative thoughts about self, and suicidal thoughts or behaviors has increased since early 2010s [1–4]. This trend coincided with dramatic growth in the use of social media [5]. Several cross-sectional, longitudinal and experimental studies have found evidence linking psychological symptoms with excessive social media use [e.g., 6,7–11]. This literature has also been extensively reviewed in past meta-analyses [8, 12–16]. The link appears to be especially strong for problematic or addictive social media use [12, 15], in which users forgo other important social and academic activities to engage in social media interactions and experience craving and withdrawal when not using these media [17].

Although a number of investigators have speculated on the potential role of increased social media use in the trends of psychological symptoms among children and adolescents in recent years [18], few studies have directly examined these associations [19, 20].

This report uses data from the Health Behaviour in School-aged Children (HBSC) survey to examine trends in psychological symptoms in adolescents in the years between 2001 and 2018—the period of introduction and rapid growth in the use of social media in adolescents. The study further examines the relationship between frequent and problematic social media use over this period with psychological symptoms.

Several past studies have examined temporal trends and correlates of mental health outcomes using HBSC, with somewhat mixed results [21–26]. A study based on all participating countries recorded only a small increase in average symptoms over time [24]. Whereas, research from individual HBSC country sites has recorded a disproportionately higher increase in more severe symptoms [27] and in older adolescent girls [25, 26]. The present study differs from past research by focusing on trends in mild, moderate or severe psychological symptoms separately by age and sex across all participating countries.

Studies have also examined association of social media use with mental health outcomes in HBSC [28–30]. The present study compares severe psychological symptoms between two periods, one before the introduction of current social media platforms (2001–2002) [31] and, another, after their widespread use (2017–2018). Lastly, the causal direction between social media variables and psychological symptoms are explored using the novel method of Linear Non-Gaussian Acyclic Models (LiNGAM) using HBSC 2017–2018 data [32].

## Methods

### Sample

The HBSC survey and its methods have been described in more detail elsewhere [33]. Briefly, HBSC is a cross-national survey sponsored by participating countries and conducted in partnership with the World Health Organization. The survey is conducted every 4 years to monitor the health behaviors of adolescents aged 11–15 across 47 countries in Europe, North America, Middle East and Central Asia (Online Resource 1) [33].

The survey uses a standardized research protocol across countries and over time, allowing for pooling the data. Stratified random cluster sampling is used with primary sampling unit defined at the level of schools in some countries and classes within schools in other countries. Participants complete anonymous questionnaires in classroom settings. Questionnaires were translated from English into national languages with back-translation and comparison of the back translated versions with original English by independent experts, following a standard protocols [33]. Institutional ethical approval in each participating country and participating schools as well as informed consent from parents and adolescents were obtained by HBSC investigators.

Data from five rounds of HBSC (2001–2002, 2005–2006, 2009–2010, 2013–2014, 2017–2018) were used in this study for assessing trend, from two rounds (2001–2002 and 2017–2018) for examining association between social media use and psychological symptoms and from the 2017–2018 round for LiNGAM analyses.

## Measurements

### Psychological symptoms

Adolescent psychological symptoms were measured using HBSC Symptom Checklist (HBSC-SC), a brief validated measure of psychological and somatic symptoms in adolescents [34]. Past factor analysis has verified the two-factor structure of HBSC-SC: a psychological symptom factor and a somatic factor, each with 4 items [35]. In this study the psychological symptoms were used which included questions about the frequency of feeling low, irritability or bad temper, feeling nervous, and difficulties in getting to sleep over the past 6 months. For each question, frequency was reported on a scale from “rarely or never” ( = 0) to “about every day” ( = 4). A summary score was computed based on these responses (score range: 0–16). The scale had adequate internal consistency in this sample (Cronbach alpha = 0.74).

### Frequency of social media use

Frequency of social media use was measured using four questions about frequency of online contact with close friends, friends from a larger friend group, online friends, and other people (e.g., parents, siblings, classmates, teachers). Responses ranged from “never or almost never” (0) to “all the time” (4). The items were not expected to be correlated as being online all the time with one group of contacts would reduce the likelihood of being online with other contacts. As such, consistent with past research [28], no summary measure was computed. Instead, the maximum frequency of use across the four items was computed (range: 0–4).

### Problematic social media use

Problematic or addictive social media use was assessed using the validated nine-item Social Media Disorder Scale [36] which assessed past-year symptoms of preoccupation with social media, withdrawal and tolerance, neglect of other activities, use of social media to cope with distress, inability to cut-down on use of social media, lying about the extent of social media use and trouble in interpersonal relationships because of it. Responses to each item are in yes ( = 1) or no ( = 0) format. The scores are summed to create a total score (range = 0–9). The items were moderately to strongly correlated (tetrachoric correlations range: 0.39–0.64) and the scale had adequate internal consistency (KR20 = 0.77). A score of ≥ 5 has been proposed for defining problematic use or social media disorder [36].

Analyses additionally adjusted for self-reported sex (male/female) and age.

## Analytic approach

Analyses were conducted in 3 stages. First, trends in psychological symptoms across the five waves of HBSC in all participants and across sex and age groups were examined. Because past research based on HBSC suggested that temporal trends in symptoms may be more pronounced for more severe symptoms [27], psychological symptoms were categorized into 4 mutually exclusive categories of severity based on symptom scores: 0–3, 4–7, 8–11 and 12–16. The models adjusted for the fixed effect of country.

Second, a series of hierarchical multinomial logistic regression analyses were conducted to examine whether adding the variables of frequency of social media use or problematic use to the models could reduce the magnitude of the regression coefficient for the survey wave variable (i.e., 2017–2018 vs. the 2001–2002 period). The 2001–2002 survey predated the introduction of all major social media platforms (e.g., Facebook in 2004, Twitter in 2006, Instagram in 2010, Pinterest in 2010, Snapchat in 2011, TikTok in 2016). Thus, although questions about social media were not asked in this survey wave, participants were assumed to have never used social media and not to meet any of the problematic social media use criteria ( = 0 on both variables).

Variables were added at each level of the hierarchical analysis and change in the regression coefficient associated with survey wave (HBSC 2001–2002 = 0 and HBSC 2017–2018 = 1) after adding the new variables was examined. The outcome of interest in these models was psychological symptoms categorized into four categories (0–3, 4–7, 8–11 and 12–16). Model 1 only adjusted for the fixed effect of country; sex and age were added in model two. The variables of frequency of social media use and problematic use of social media were each added separately in the third and fourth models. Because the fifth model with both frequency of social media use and problematic use of social media produced results very similar to the model with problematic social media use, only the results of the first four models are presented here.

Multiple imputations using chained equations [37] with five imputed datasets were used to impute missing data in hierarchical regression analyses. Complete case analyses were also conducted as a sensitivity analysis. In further sensitivity analysis, participants for the hierarchical analyses were limited to 29 countries that were surveyed in both 2001–2002 and 2017–2018.

Analyses of trends and the hierarchical regression models adjusted for survey weights and other survey elements. The survey commands of Stata 18 (StataCorp, LLC, College Station, TX, 2023) were used for these analyses. All percentages reported are weighted unless indicated otherwise. A conservative *p*-value of < 0.01 was used to determine statistical significance.

LiNGAM was used to explore causal direction suggested by the data. LiNGAM is based on the assumption that in the regression model with a correctly specified causal direction, the putative cause and the error term are independent. Whereas, in the incorrectly specified model the two are not independent. To be able to suggest a direction, the distribution of at least one of the variables needs to deviate from normality. If both the putative cause and the putative effect are normally distributed, the causal direction cannot be inferred. LiNGAM is based on the strong assumption of no confounding, which cannot be confirmed given the cross-sectional nature of the data. Additionally, LiNGAM assumes a linear relationship between the two variables. Although, as Shimuzu notes, linear relationships almost never exist in the real world [38]. But, in general, linear models provide better results in comparison to non-linear models for exploring the direction of causality [38]. The R Implementation of the DirectLiNGAM algorithms [39] in the *rlingam* package by Genta Kikuchi (https://github.com/gkikuchi/rlingam) was used. Both social media variables and psychological symptoms were standardized to range from 0 to 1 for the LiNGAM analyses. A more detailed description of the DirectLiNGAM is provided in Online Resource 2.

Causal direction was tested in 1000 bootstrapped replications. Mean and confidence intervals of the LiNGAM regression coefficients from these bootstrapped replicates were computed [40].

As a sensitivity analysis, three sets of further LiNGAM analyses with simulated data were conducted in which variables of frequency of social media use, problematic social media use and psychological symptoms were included as independent variables (cause) and dependent variables (effect) were simulated for each using ordinary least square. Sensitivity analyses then sought to examine whether LiNGAM could detect the correct causal direction among these causes and the simulated effects.

## Results

A total of 1,036,869 adolescents participated in the five HBSC surveys. Breakdown of the sample by country is presented in Online Resource 1. Of these, 985,441 (95.0% unweighted) responded to the psychological symptoms, age and sex questions and comprised the sample for the trend analyses. The average age of these participants was 13.6 (standard deviation [SD] = 1.6) years and 51.2% were female. The mean of psychological symptom score in the sample was 4.9 (SD = 4.0).

**Fig. 1:**
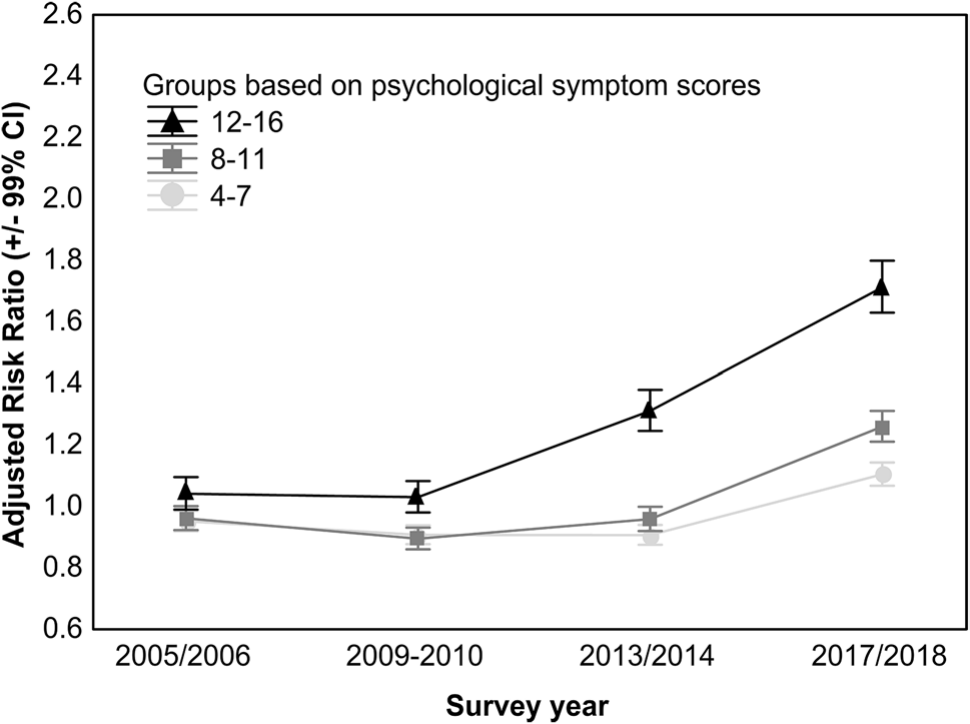
Temporal trends in psychological symptoms across the 2001–2002 to 2017–2018 waves of Health Behaviour in School-aged Children survey. Adjusted risk ratios are derived from multinomial logistic regression analyses with outcome categories based on the levels of psychological symptoms (4–7, 8–11 and 12–16, with the 0–3 score category as the reference) (Online Resource 3). Each survey wave was compared with the 2001–2002 survey wave. Models adjusted for the fixed effect of country, for survey weights, stratification, and clustering

The mean psychological symptom score increased modestly from 4.74 in 2001–2002 to 5.32 in 2017–2018—a change of approximately 0.15 standard deviations. However, the change was not even across levels of severity. For example, while the risk for symptoms in the 4–7 score category increased by 9% (adjusted risk ratio [ARR] = 1.09, 99% confidence interval [CI] 1.06–1.13), the risk for symptoms in the 12–16 score category increased by 66% (ARR = 1.66, 99% CI 1.57–1.74; Online Resource 3). Risk ratios from multinomial logistic regression analysis for comparison of each psychological symptom level against the 0–3 symptom level category are presented in Fig. 1.

**Fig. 2:**
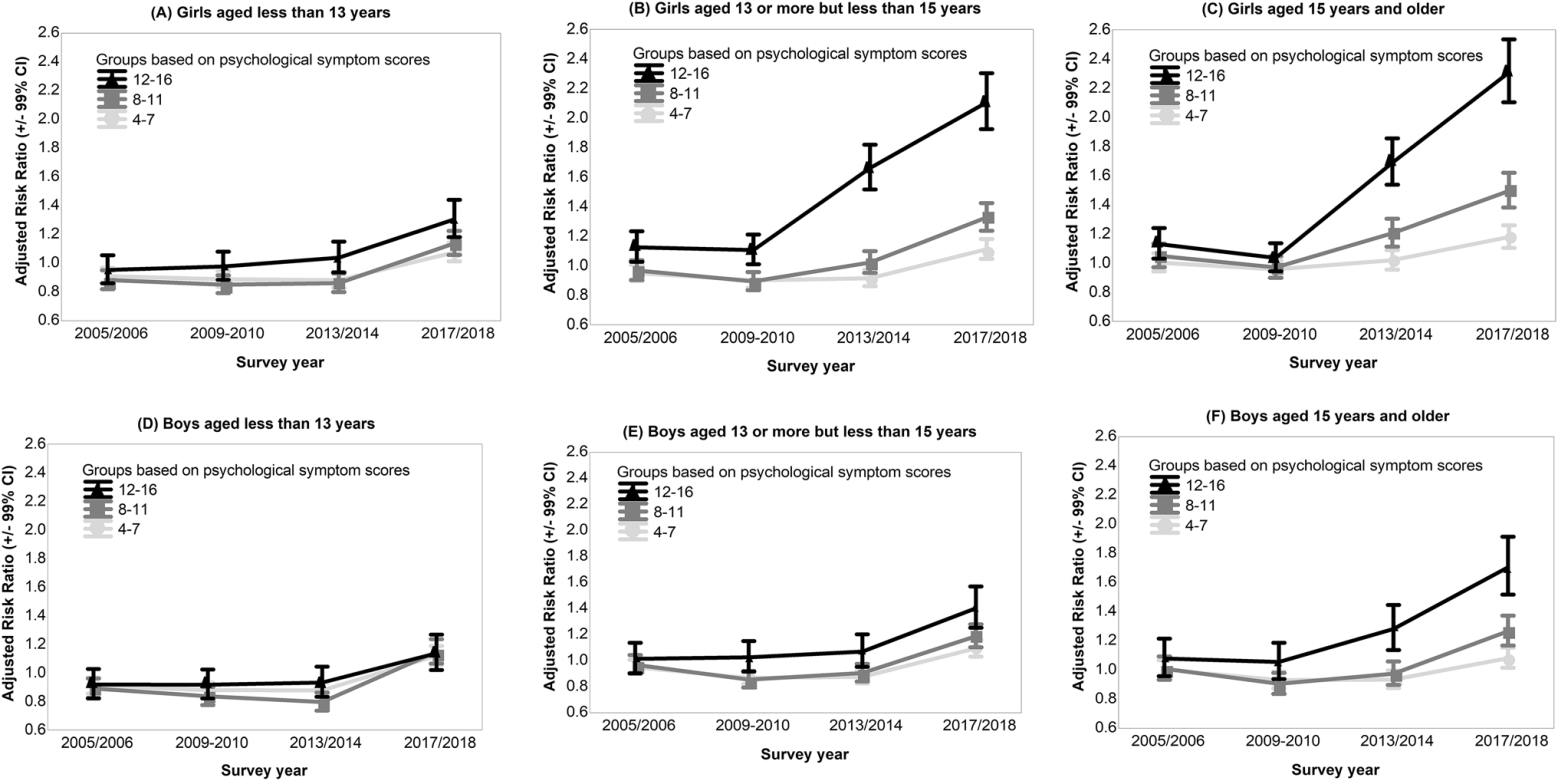
**A-F** Temporal trends in psychological symptoms across the 2001–2002 to 2017–2018 waves of Health Behaviour in School-aged Children survey according to sex and age group. Adjusted risk ratios are derived from multinomial logistic regression analyses with outcome categories based on levels of psychological symptoms (4–7, 8–11 and 12–16, with the 0–3 score category as the reference category) (Online Resource 3). Each survey wave was compared with the 2001–2002 survey wave. Models adjusted for the fixed effect of country, for survey weights, stratification, and clustering

The time trend across symptom levels was most pronounced among older adolescent girls (Fig 2A-F; Online Resource 3). For example, while the risk of symptoms in the 12–16 score category increased only by 14% in boys < 13 (ARR = 1.14, 99% CI 1.02–1.27) it more than doubled in girls > 15 (ARR = 2.31, 99% CI 2.10–2.53) (Online Resource 3).

**Fig. 3:**
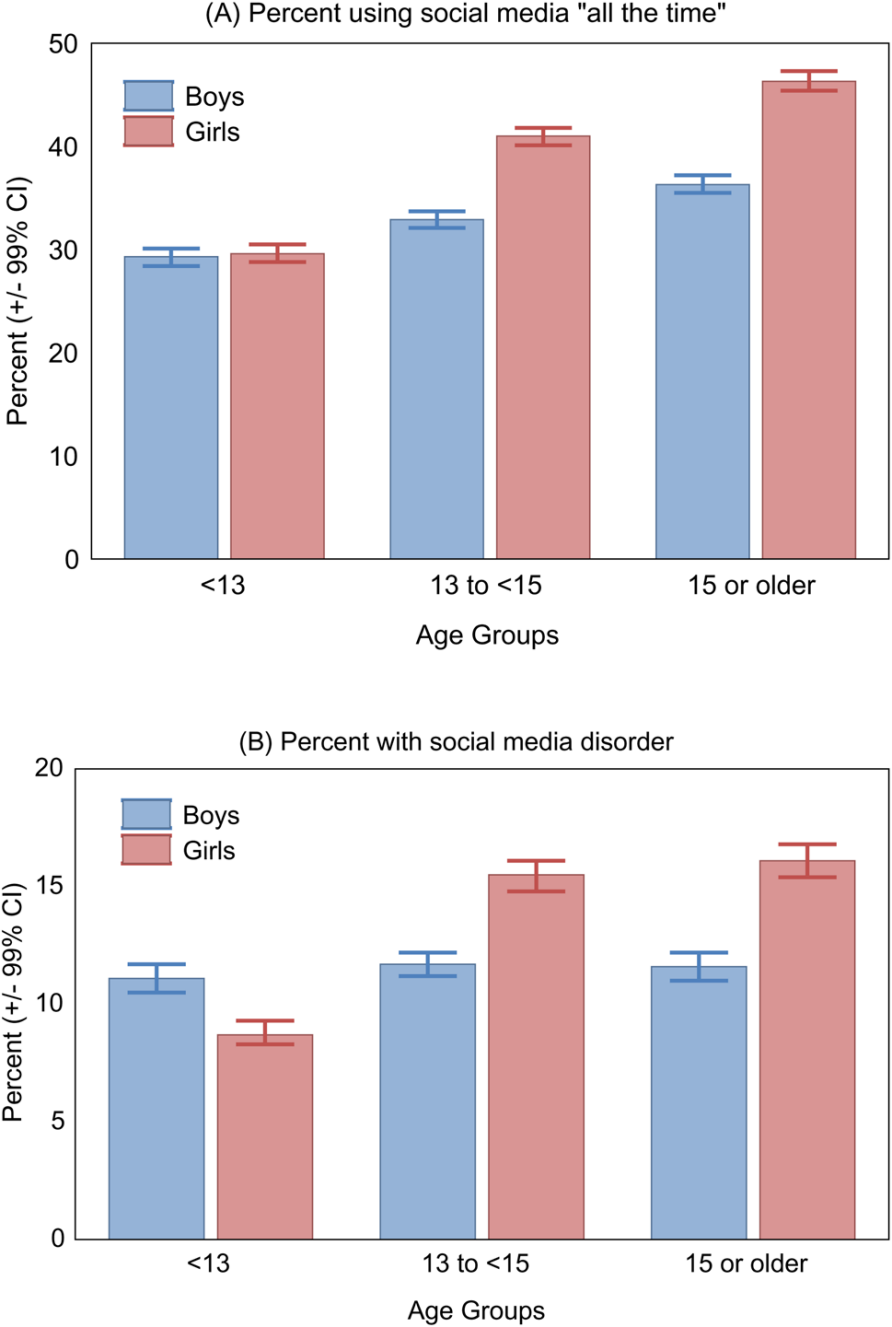
**A-B** Percent of participants of the 2017–2018 Health Behaviour in School-aged Children survey who reported using social media “all the time” (**A**) or reported problematic social media use at a level to qualify for social media disorder ( ≥ 5 on Social Media Disorder Scale[36]), according to sex and age

The mean score on the social media frequency of use scale in 2017–2018 was 2.8 (SD = 1.2); 36.0% of adolescents reported being on social media “all the time” (score = 4). The mean score on the problematic social media use scale was 1.8 (SD = 2.1), with 12.5% scoring in the social media disorder range (score ≥ 5) [36]. The sex and age pattern of frequency of social media use and social media disorder paralleled the patterns in psychological symptoms, with older adolescent girls being more likely to report using social media all the time and to meet criteria for social media disorder (Figure 3A-B, Online Resource 4).

Hierarchical multinomial logistic regression analyses that were based on data from HBSC 2001–2002 and 2017–2018 (n = 403,256) and adjusted for country, produced similar results as the main trend analysis, indicating significantly higher prevalence of the more severe psychological symptoms in the later period (ARR = 1.72, 99% CI 1.64–1.81; Table 1). Adjusting for sex and age did not modify the effect of survey wave appreciably (Table 1). However, entering the variable of frequency of social media use in the model reduced the coefficient for survey wave (ARR = 1.18, 99% CI 1.10–1.28). After entering the variable of problematic social media use, the regression coefficient for survey wave was reduced to less than one (ARR = 0.79, 99% CI 0.75–0.84, *p* < 0.001, Table 1). Both variables of frequency of social media use and problematic use of social media were significantly associated with psychological symptoms, especially more severe symptoms (ARR = 1.16, 99% CI 1.13–1.18, *p* < 0.001, and ARR = 1.48, 99% CI 1.47–1.50, *p* < 0.001, respectively) (Table 1).

**Table 1:** Results of hierarchical multinomial logistic regression analyses of the association of survey waves (2001–2002 vs. 2017–2018) with psychological symptoms in participants of Health Behaviour in School-aged Children survey

	Psychological symptom score categories (compared with 0–3 category)								
	4–7			8–11			12–16		
	ARR	99% CI	*p*	ARR	99% CI	*p*	ARR	99% CI	*p*
Model not adjusting for individual characteristics									
Year									
2001–2002	1.00	Ref.	–	1.00	Ref.	–	1.00	Ref.	–
2017–2018	1.09	1.06–1.12	< 0.001	1.24	1.20–1.29	< 0.001	1.72	1.64–1.81	< 0.001
Model adjusting for sex and age									
Year									
2001–2002	1.00	Ref.	–	1.00	Ref.	–	1.00	Ref.	–
2017–2018	1.10	1.07–1.13	< 0.001	1.27	1.22–1.31	< 0.001	1.78	1.70–1.87	< 0.001
Sex									
Female	1.00	Ref.	–	1.00	Ref.	–	1.00	Ref.	–
Male	0.75	0.74–0.77	< 0.001	0.54	0.53–0.56	< 0.001	0.39	0.38–0.41	< 0.001
Age, years	1.11	1.10–1.12	< 0.001	1.17	1.16–1.18	< 0.001	1.25	1.24–1.27	< 0.001
Model adjusting for sex, age, and frequency of social media use									
Year									
2001–2002	1.00	Ref.	–	1.00	Ref.	–	1.00	Ref.	–
2017–2018	0.98	0.94–1.03	0.272	1.00	0.95–1.05	0.994	1.18	1.10–1.28	< 0.001
Sex									
Female	1.00	Ref.	–	1.00	Ref.	–	1.00	Ref.	–
Male	0.76	0.74–0.77	< 0.001	0.55	0.54–0.56	< 0.001	0.40	0.38–0.41	< 0.001
Age, years	1.10	1.10–1.11	< 0.001	1.16	1.15–1.17	< 0.001	1.24	1.22–1.25	< 0.001
Frequency of social media use	1.04	1.03–1.06	< 0.001	1.09	1.07–1.11	< 0.001	1.16	1.13–1.18	< 0.001
Model adjusting for sex, age, and problematic social media use									
Year									
2001–2002	1.00	Ref.	–	1.00	Ref.	–	1.00	Ref.	–
2017–2018	0.85	0.82–0.88	< 0.001	0.75	0.72–0.78	< 0.001	0.79	0.75–0.84	< 0.001
Sex									
Female	1.00	Ref.	–	1.00	Ref.	–	1.00	Ref.	–
Male	0.76	0.74–0.78	< 0.001	0.55	0.54–0.57	< 0.001	0.40	0.39–0.42	< 0.001
Age, years	1.10	1.09–1.11	< 0.001	1.15	1.14–1.16	< 0.001	1.23	1.21–1.24	< 0.001
Problematic social media use	1.18	1.17–1.19	< 0.001	1.34	1.33–1.35	< 0.001	1.48	1.47–1.50	< 0.001

Missing data were multiply imputed,* ARR* stands for adjusted risk ratio, *CI* for confidence intervals from multinomial logistic regression models in which psychological symptom scores were the outcome of interest and survey wave, the independent variable of interest. Models additionally adjusted for the fixed effect of country (not shown) and for survey weights, stratification, and clustering

Results were quite similar in complete case analyses (Online Resource 5) and in analysis limited to countries participating in both 2001–2002 and 2017–2018 surveys (Online Resource 6).

Before conducting the LiNGAM analyses, deviation from normality of the distribution of the social media variables and psychological symptoms and linear relationship were tested. The relationship between frequency of social media use and psychological symptoms was mostly linear (Online Resource 7), although the quadratic term was significant in regression analysis (regression coefficient for quadratic term = 0.120, standard error [SE] = 0.008, *p* < 0.001). Similarly, the relationship between problematic use of social media and psychological symptoms was linear across most of the range of scores with minor deviation from linearity at extreme values (Online Resource 8) (coefficient for quadratic term = 0.073, SE = 0.005, *p* < 0.001). The distribution of all three variables was highly skewed, significantly deviating from normality based on the Kolmogorov-Smirnov test (*p* < 0.001 for all three variables).

The results of DirectLiNGAM analysis suggested a direction from frequency of social media use to psychological symptoms. This result was confirmed in all 1000 bootstrap replications (mean regression coefficient = 0.090, 95% CI 0.086–0.094). Similarly, the suggested direction of effect was consistently from problematic use of social media to psychological symptoms in all of the 1000 bootstrap replications (mean regression coefficient = 0.333, 95% CI 0.328–0.339).

In sensitivity analyses, the simulated “effect” variables created were correctly detected in the LiNGAM analyses in all simulations for frequency of social media use and for problematic use as well as for psychological symptoms.

## Discussion

There were three main findings in this study. First, the prevalence of more severe psychological symptoms among adolescents appears to have increased in the past two decades. This increase was especially pronounced among adolescent girls over age 15—the group using social media most frequently and being most likely to experience problematic use.

Second, the higher prevalence of severe psychological symptoms in 2017–2018 compared to 2001–2002 period disappeared after taking account of problematic social media use. In the model adjusting for this variable, the risk ratio for survey wave was less than one. This could suggest that in the absence of problematic social media use, adolescents would have had fewer psychological symptoms in 2017–2018 compared to 2001–2002. This explanation is consistent with the results of past research suggesting that social media use is a risk factor for increased prevalence of psychological symptoms [6–16]. The alternative explanation of the study’s findings is that the increase in psychological symptoms over time has led children and adolescents to problematic use of social media. Yet another explanation is that a third factor caused both an increase in severe psychological problems in recent years and problematic social media use in the same group of children and adolescents who experienced the increase in symptoms. Lastly, in some cases, the causation may be reciprocal as suggested by some past research [41–43].

Third, the results of DirectLiNGAM analysis are consistent with the direction of effect being from frequent and problematic use of social media to psychological symptoms and not vice versa. As noted, these analyses are based on the strong assumption of no confounding.

In interpreting the results of this study its limitations should be considered. First, this report focused on the negative effects of frequent or problematic use of social media. Social media use in moderation may have beneficial effects for some adolescents [44]. Second, data on social media use for 2001–2002 were inferred, not directly measured. However, this inference is based on the fact that all major social media platforms were introduced in subsequent years and very few adolescents could be using social media in the 2001–2002 period. Although they may have been engaging in other forms of screen activity, they were not exposed to the specific effects of social media. Nevertheless, the findings do not confer the same level of certainty as a randomized controlled trials given the possibility of confounding by unmeasured variables. However, randomized trials beyond brief social media holidays are not feasible given the widespread use of these media among adolescents. Third, the LiNGAM analyses are based on the strong assumption of no confounding, an assumption that could not be tested given the cross-sectional nature of the data. Also because of the cross-sectional nature of the data, change in adolescents’ mental health as a result of change in social media use could not be examined.

## Conclusions

In conclusion and in the context of these limitations, the results of this study are in line with past longitudinal, quasi-experimental and short-term experimental studies suggesting that excessive and problematic use of social media may have a detrimental effect on the mental health of adolescents. These concerns led to a recent advisory by US Surgeon General which calls attention to the mental health harms associated with excessive and problematic use of social media as an “urgent public health issue” [45]. Interventions by parents and families to limit social media use, as well as policies to limit use of algorithms that are conducive to problematic use of these media may help reduce their negative mental health impact in the coming years.

## Supplementary Information

Below is the link to the electronic supplementary material.

Supplementary file1 (PDF 421 KB)
