# Correction: Problematic social media use and psychological symptoms in adolescents

**DOI:** 10.1007/s00127-025-02973-6

**Published:** 2025-08-18

**Authors:** Ramin Mojtabai

**Affiliations:** Department of Psychiatry and Behavioral Sciences, Tulane University, 1440 Canal Street, Suite 1000, New Orleans, LA 70112 USA

**Correction to: Social Psychiatry and Psychiatric Epidemiology (2024) 59:2271–2278**

10.1007/s00127-024-02657-7

The Conflict-of-Interest section was missing from this article and should have read ‘Dr. Mojtabai has received royalties and consulting fees from UpToDate, Medscape and MindMed, and provided consultation regarding social media litigation on behalf of the Plaintiffs’.

The original article has been corrected.

