# Supplementary material for: Problematic social media use and psychological symptoms in adolescents
Source: Soc Psychiatry Psychiatr Epidemiol. 2024 Apr 7;59(12):2271–8. doi: 10.1007/s00127-024-02657-7 (PMC11522145; doi:10.1007/s00127-024-02657-7)

Online Resources for “Problematic Social Media Use and Psychological Symptoms in Adolescents” *Social Psychiatry and Psychiatric Epidemiology*

Ramin Mojtabai, MD, PhD, MPH

Department of Psychiatry and Behavioral Sciences, Tulane University, New Orleans, LA (email: rmojtabai@tulane.edu)

Online Resource 1: Number of participants of Health Behaviours in School-aged Children, 2001-2018 according to country.

Country	Survey year					Total
	2001-2002	2005-2006	2009-2010	2013-2014	2017-2018	
Albania	0	0	0	5,024	1,765	6,789
Azerbaijan	0	0	0	0	4,586	4,586
Austria	4,472	4,848	5,043	3,458	4,129	21,950
Armenia	0	0	2,833	3,679	4,717	11,229
Belgium (Flemish)	6,289	4,311	4,180	4,393	4,333	23,506
Belgium (French)	4,323	4,476	4,012	5,892	5,578	24,281
Bulgaria	0	4,854	0	4,796	4,548	14,198
Canada	4,361	5,930	15,919	12,931	12,950	52,091
Croatia	4,397	4,968	6,262	5,741	5,169	26,537
Czech Republic	5,012	4,782	4,425	5,082	11,564	30,865
Denmark	4,672	5,741	4,330	3,891	3,181	21,815
Estonia	3,979	4,484	4,236	4,057	4,725	21,481
Finland	5,388	5,249	6,723	5,925	0	23,285
France	8,185	7,155	6,160	5,691	9,170	36,361
Georgia	0	0	0	0	4,242	4,242
Germany	5,650	7,274	5,005	5,961	4,347	28,237
Greece	3,807	3,715	4,944	4,141	3,863	20,470
Greenland	891	1,366	1,207	1,020	1,243	5,727
Hungary	4,164	3,532	4,864	3,935	3,789	20,284
Iceland	0	9,540	11,119	10,602	6,996	38,257
Ireland	2,875	4,894	4,965	4,098	3,833	20,665
Israel	5,661	5,686	4,135	6,193	7,712	29,387
Italy	4,386	3,951	4,837	4,072	4,144	21,390
Kazakhstan	0	0	0	0	4,868	4,868
Latvia	3,481	4,245	4,284	5,557	4,412	21,979
Lithuania	5,645	5,632	5,338	0	3,797	20,412
Luxembourg	0	4,387	4,228	3,318	4,070	16,003
Malta	1,980	1,404	0	2,265	2,576	8,225
Republic of Moldova	0	0	0	4,648	4,686	9,334
Netherlands	4,268	4,278	4,591	4,301	4,698	22,136
Norway	5,023	4,711	4,342	3,422	3,127	20,625
Poland	6,383	5,489	4,262	4,545	5,224	25,903
Portugal	2,940	3,919	4,036	4,989	6,126	22,010
Romania	0	4,684	5,404	3,980	4,567	18,635
Russia	8,037	8,231	5,174	4,716	4,281	30,439
Serbia	0	0	0	0	3,933	3,933
Slovakia	0	3,882	5,344	6,099	4,785	20,110
Slovenia	3,956	5,130	5,436	4,997	5,667	25,186
Spain	5,827	8,891	5,040	11,136	4,320	35,214
Sweden	3,926	4,415	6,718	7,700	4,185	26,944
Switzerland	4,679	4,621	6,678	6,634	7,510	30,122
Turkey	0	5,639	5,664	0	5,848	17,151
Ukraine	4,090	5,069	5,890	4,552	6,660	26,261
Macedonia	4,161	5,281	3,944	4,218	4,658	22,262
England	6,081	4,783	3,524	5,335	3,397	23,120
Scotland	4,404	6,190	6,771	5,932	5,021	28,318
Wales	3,887	4,409	5,454	5,154	15,951	34,855
USA	5,025	3,892	6,274	0	0	15,191
**Total**	**162,305**	**205,938**	**213,595**	**214,080**	**240,951**	**1,036,869**

Online Resource 2:

Description of the Direct Linear Non-Gaussian Acyclic Model

Direct Linear Non-Gaussian Acyclic Models (DirectLiNGAM) helps choosing between the correctly specified causal mode (cause → effect) and the incorrectly specified model (effect → cause) under the assumptions that the causal effect is linear, the error terms are non-Guassian and there is no confounding.

Intuitively, in the correctly specified causal model both the predictor (cause) and the error variable (noise) are exogenous. Whereas, in the incorrectly specified model, the error term is not exogenous to the model and not independent from the predictor variable because the true causal variable is the “parent” of both.

In effect, LiNGAM distinguishes between two alternative data generating models (1) and (2) below:

$$\begin{cases} Y = aX + e_Y \\ X = e_X \end{cases} \quad (1),$$

$$\begin{cases} X = bY + \hat{e}_X \\ Y = \hat{e}_Y \end{cases} \quad (2).$$

Assuming model (1) is the correctly specified causal model, X is the true cause variable and Y is the effect variable; e_X and e_Y are the exogenous error terms. Whereas, assuming that model (2) is the true causal model, Y is the cause and X is the effect variable, with $\hat{e}_X$ and $\hat{e}_Y$ being the exogenous error terms. Independence between the putative cause and the error will hold only in one direction, i.e., X will be independent from e_Y if the data generating model in (1) is the correct model. Alternatively, Y will be independent from $\hat{e}_X$ if the data generating model in (2) is the correct model. Of note, the Pearson correlations between the error terms and the putative causes are, by design, non-significant in both models.

DirectLiNGAM is based on the Darmois-Skitovich theorem which states that if two random variables l_1 and l_2 that are the linear sums of the independent random variables, *si (i=1,…q),* depicted in (3) and (4) below, are independent, then s_i are Gaussian.[2]

$$l_1 = \sum_{i=1}^{q} \alpha_i s_i \qquad (3),$$

$$l_2 = \sum_{i=1}^{q} \beta_i s_i \qquad (4).$$

The contrapositive of this statement is that if even one s_i is non-Gaussian, then l_1 and l_2 cannot be independent. This contrapositive of the Darmois-Skitovich theorem has implications for DirectLiNGAM as it predicts that if one of the putative causal models (1) or (2) is the correct causal model, in which the predictor (true cause) and the error term are independent, the predictor and error term in the incorrect causal model would not be independent. This can be shown as follows. Assuming that model 1 is the correct causal model and rewriting (2), the incorrect model, by substituting (1), for *Y* we obtain:

$$\hat{e}_X = (1 - a.b)\, X - a.e_Y \quad (5).$$

Note that both (1) and (5) are linear combinations of the non-Gaussian variables *X* and e_Y. As such, according to the Darmois-Skitovich theorem, *Y* and $\hat{e}_X$ cannot be independent, ruling out model (2) as the causal model. Thus, identifying that the predictor and the error term are dependent in regression models (1) or (2) suggests that the model is not the correct causal model.

REFERENCES

1. Shimizu S, Hoyer PO, Hyvärinen A, Kerminen A, Jordan M (2006) A linear non-Gaussian acyclic model for causal discovery. Journal of Machine Learning Research 7 (10): 2003-2030

2. Shimizu S, Inazumi T, Sogawa Y, Hyvarinen A, Kawahara Y, Washio T, Hoyer PO, Bollen K, Hoyer P (2011) DirectLiNGAM: A direct method for learning a linear non-Gaussian structural equation model. Journal of Machine Learning Research-JMLR 12 (Apr): 1225-1248

3. Eberhardt F (2017) Introduction to the foundations of causal discovery. International Journal of Data Science and Analytics 3: 81-91.

Online Resource 3: Results of the analyses for temporal trends in psychological symptoms across the 2001-2002 to 2017-2018 waves of Health Behaviour in School-aged Children survey according to sex and age groups. Adjusted risk ratios are derived from multinomial logistic regression analyses with categories based on levels of psychological symptoms as the outcome (4-7, 8-11 and 12-16, with 0-3 category as reference). Each survey wave was compared with the 2001-2002 survey wave.

	**Psychological symptom score categories**												
	**0-3**	**4-7**				**8-11**				**12-16**			
**Sex & Age group/ Survey wave**	**%**	**%**	**ARR**	**99% CI**	***p***	**%**	**ARR**	**99% CI**	***p***	**%**	**ARR**	**99% CI**	***p***
Boys <13 yrs.													
2001-2002	53.1	28.8	1.00	Ref.	--	13.2	1.00	Ref.	--	4.9	1.00	Ref.	--
2005-2006	54.7	27.5	0.91	0.86-0.97	<.001	12.7	0.89	0.83-0.96	<.001	5.1	0.92	0.82-1.03	.051
2009-2010	55.1	27.5	0.88	0.83-0.93	<.001	12.3	0.84	0.78-0.90	<.001	5.1	0.92	0.82-1.02	.042
2013-2014	56.5	26.8	0.88	0.83-0.93	<.001	11.4	0.80	0.74-0.86	<.001	5.2	0.93	0.83-1.04	.110
2017-2018	49.6	29.6	1.12	1.06-1.19	<.001	14.7	1.15	1.06-1.24	<.001	6.0	1.14	1.02-1.27	.002
Boys 13 to <15 yrs.													
2001-2002	50.3	31.3	1.00	Ref.	--	13.6	1.00	Ref.	--	4.8	1.00	Ref.	--
2005-2006	50.6	30.4	0.95	0.90-1.01	.024	13.7	0.97	0.90-1.04	.278	5.2	1.01	0.91-1.14	.740
2009-2010	52.1	29.5	0.87	0.82-0.92	<.001	12.8	0.86	0.79-0.92	<.001	5.6	1.03	0.92-1.15	.552
2013-2014	52.8	28.9	0.88	0.83-0.93	<.001	12.9	0.91	0.84-0.98	.001	5.4	1.07	0.95-1.20	.130
2017-2018	46.3	31.4	1.09	1.03-1.16	<.001	15.6	1.19	1.10-1.28	<.001	6.7	1.40	1.25-1.57	<.001
Boys ≥15													
2001-2002	47.3	33.1	1.00	Ref.	--	14.7	1.00	Ref.	--	4.9	1.00	Ref.	--
2005-2006	46.3	32.8	1.00	0.94-1.07	.877	15.1	1.01	0.93-1.09	.749	5.7	1.08	0.96-1.22	.097
2009-2010	48.1	32.0	0.93	0.88-0.99	.002	14.1	0.91	0.84-0.98	.001	5.8	1.06	0.94-1.19	.240
2013-2014	47.7	31.2	0.94	0.88-1.00	.008	14.5	0.98	0.90-1.06	.431	6.5	1.28	1.14-1.45	<.001
2017-2018	43.4	31.4	1.08	1.01-1.15	.002	16.9	1.27	1.17-1.37	<.001	8.3	1.70	1.52-1.91	<.001
Girls <13 yrs.													
2001-2002	48.3	30.0	1.00	Ref.	--	15.4	1.00	Ref.	--	6.3	1.00	Ref.	--
2005-2006	50.1	28.5	0.91	0.86-0.97	<.001	14.7	0.88	0.82-0.95	<.001	6.7	0.95	0.86-1.06	.232
2009-2010	50.0	28.6	0.89	0.84-0.94	<.001	14.5	0.85	0.79-0.92	<.001	7.0	0.98	0.89-1.08	.564
2013-2014	51.6	27.5	0.88	0.83-0.94	<.001	14.1	0.86	0.80-0.93	<.001	6.9	1.04	0.94-1.15	.341
2017-2018	45.9	29.2	1.08	1.01-1.14	.002	16.7	1.14	1.06-1.23	<.001	8.3	1.31	1.18-1.44	<.001

Girls 13 to <15 yrs.													
2001-2002	38.5	33.4	1.00	Ref.	--	20.0	1.00	Ref.	--	8.0	1.00	Ref.	--
2005-2006	38.5	31.9	0.95	0.90-1.01	.024	19.9	0.97	0.90-1.04	.216	9.7	1.12	1.03-1.23	.001
2009-2010	39.1	31.6	0.90	0.85-0.95	<.001	19.2	0.89	0.83-0.96	<.001	10.0	1.11	1.01-1.21	.004
2013-2014	38.0	29.4	0.92	0.86-0.97	<.001	19.8	1.02	0.95-1.10	.433	12.8	1.66	1.52-1.82	<.001
2017-2018	33.1	30.1	1.11	1.05-1.18	<.001	22.2	1.33	1.24-1.42	<.001	14.6	2.11	1.92-2.30	<.001
Girls ≥15 yrs.													
2001-2002	31.7	34.8	1.00	Ref.	--	22.6	1.00	Ref.	--	10.9	1.00	Ref.	--
2005-2006	30.3	33.6	1.00	0.94-1.07	.879	23.6	1.05	0.97-1.13	.108	12.6	1.13	1.03-1.24	.001
2009-2010	31.6	33.6	0.96	0.90-1.02	.069	22.8	0.97	0.90-1.05	.316	12.0	1.04	0.94-1.14	.330
2013-2014	28.2	31.1	1.02	0.95-1.09	.447	24.5	1.20	1.11-1.30	<.001	16.2	1.69	1.54-1.86	<.001
2017-2018	25.3	30.0	1.18	1.10-1.26	<.001	25.6	1.50	1.38-1.62	<.001	19.1	2.31	2.10-2.53	<.001
**Total**													
**2001-2002**	**45.0**	**31.8**	**1.00**	**Ref.**	**--**	**16.6**	**1.00**	**Ref.**	**--**	**6.7**	**1.00**	**Ref.**	
**2005-2006**	**45.0**	**30.8**	**0.96**	**0.93-0.99**	**.001**	**16.7**	**0.97**	**0.93-1.01**	**.048**	**7.5**	**1.05**	**1.00-1.11**	**.007**
**2009-2010**	**45.9**	**30.5**	**0.91**	**0.88-0.94**	**<.001**	**16.0**	**0.90**	**0.86-0.93**	**<.001**	**7.6**	**1.03**	**0.98-1.08**	**.139**
**2013-2014**	**45.8**	**29.1**	**0.91**	**0.88-0.94**	**<.001**	**16.3**	**0.96**	**0.92-1.00**	**.010**	**8.9**	**1.31**	**1.24-1.38**	**<.001**
**2017-2018**	**40.8**	**30.3**	**1.09**	**1.06-1.13**	**<.001**	**18.6**	**1.23**	**1.18-1.28**	**<.001**	**10.4**	**1.66**	**1.57-1.74**	**<.001**

Abbreviations: ARR stands for adjusted risk ratios and CI for confidence intervals from multinomial logistic regression models. Separate analyses were conducted for sex/age groups. Models adjusted for the fixed effect of country (not shown) and for survey weights, stratification, and clustering in classroom.

Online Resource 4: Prevalence of frequent social media use and social media disorder in participants of Health Behaviour in School-aged Children, 2017-2018 survey according to sex and age as well as multinomial logistic regression results for comparing each sex/age category with boys aged less than 13 years.

	**Using social media "all the time"[a]**					**Social media disorder (problematic social media use)[b]**				
**Age/sex group**	**Percent**	**99% CI**	**AOR[c]**	**99% CI**	***p***	**Percent**	**99% CI**	**AOR**	**99% CI**	***p***
Boys, <13 yrs.	29.3	28.4-30.1	1.00	Ref.	--	11.1	10.5-11.7	1.00	Ref.	--
Boys, 13 to <15 yrs.	32.9	32.1-33.7	1.20	1.14-1.26	<.001	11.7	11.2-12.2	1.08	1.004-1.17	.007
Boys, ≥15 yrs.	36.3	35.5-37.2	1.40	1.33-1.48	<.001	11.6	11.0-12.2	1.07	0.99-1.16	.033
Girls, <13 yrs.	29.6	28.8-30.5	1.01	0.96-1.06	.513	8.7	8.3-9.3	0.76	0.71-0.82	<.001
Girls, 13 to <15 yrs.	41.0	40.1-41.8	1.72	1.63-1.81	<.001	15.5	14.8-16.1	1.49	1.39-1.60	<.001
Girls, ≥15 yrs.	46.3	45.4-47.3	2.14	2.02-2.26	<.001	16.1	15.4-16.8	1.56	1.45-1.68	<.001

a. Based on reports of frequency of online contact with close friends, friends from a larger friend group, online friends or other people (e.g., parents, siblings, classmates, teachers)
b. Based on a score of ≥5 on the Social Media Disorder Scale (Van den Eijnden RJ, Lemmens JS, Valkenburg PM. The social media disorder scale. *Computers in Human Behavior* 2016; 61: 478-87).
c. Based on a binary logistic regression model that adjusted for country fixed effects.

Online Resource 5: Results of hierarchical multinomial logistic regression analyses of the association of survey waves (2001-2002 vs. 2017-2018) and psychological symptoms in participants of Health Behaviour in School-aged Children survey. Complete case analysis.

	**Psychological symptom score categories (compared with 0-3 category)**								
	**4-7**			**8-11**			**12-16**		
	**ARR**	**99% CI**	***p***	**ARR**	**99% CI**	***p***	**ARR**	**99% CI**	***p***
**Model not adjusting for individual characteristics[a]**									
Year									
2001-2002	1.00	Ref.	--	1.00	Ref.	--	1.00	Ref.	--
2017-2018	1.09	1.06-1.12	<.001	1.24	1.20-1.29	<.001	1.72	1.64-1.81	<.001
**Model adjusting for sex and age[b]**									
Year									
2001-2002	1.00	Ref.	--	1.00	Ref.	--	1.00	Ref.	--
2017-2018	1.10	1.07-1.13	<.001	1.27	1.22-1.31	<.001	1.78	1.70-1.87	<.001
Sex									
Female	1.00	Ref.	--	1.00	Ref.	--	1.00	Ref.	--
Male	0.75	0.74-0.77	<.001	0.54	0.53-0.56	<.001	0.39	0.38-0.41	<.001
Age, years	1.11	1.10-1.12	<.001	1.17	1.16-1.18	<.001	1.25	1.24-1.27	<.001
**Model adjusting for sex, age, and frequency of social media use[c]**									
Year									
2001-2002	1.00	Ref.	--	1.00	Ref.	--	1.00	Ref.	--
2017-2018	1.03	0.99-1.07	.024	1.09	1.04-1.14	<.001	1.32	1.24-1.40	<.001
Sex									
Female	1.00	Ref.	--	1.00	Ref.	--	1.00	Ref.	--
Male	0.75	0.73-0.77	<.001	0.54	0.53-0.56	<.001	0.39	0.37-0.40	<.001
Age, years	1.10	1.10-1.11	<.001	1.16	1.15-1.17	<.001	1.24	1.23-1.26	<.001
Frequency of social media use	1.05	1.03-1.06	<.001	1.09	1.07-1.11	<.001	1.17	1.14-1.19	<.001
**Model adjusting for sex, age, and problematic social media use[d]**									
Year									
2001-2002	1.00	Ref.	--	1.00	Ref.	--	1.00	Ref.	--
2017-2018	0.85	0.83-0.87	.001	0.75	0.73-0.77	<.001	0.79	0.76-0.83	<.001
Sex									
Female	1.00	Ref.	--	1.00	Ref.	--	1.00	Ref.	--
Male	0.75	0.74-0.77	<.001	0.54	0.53-0.55	<.001	0.39	0.37-0.40	<.001
Age, years	1.10	1.10-1.11	<.001	1.15	1.14-1.16	<.001	1.22	1.21-1.23	<.001
Problematic social media use	1.19	1.19-1.20	<.001	1.35	1.34-1.36	<.001	1.49	1.47-1.50	<.001

Abbreviations: ARR stands for adjusted risk ratio, CI for confidence intervals from multinomial logistic regression models in which psychological symptom scores were the outcome of interest and survey wave, the independent variable of interest. Models additionally adjusted for the fixed effect of country (not shown) and for survey weights, stratification, and clustering in classroom.

a. n=386,366.

b. n=383,752.

c. n=350,275.

d. n=346,661.

Online Resource 6: Results of hierarchical multinomial logistic regression analyses of the association of survey waves (2001-2002 vs. 2017-2018) and psychological symptoms in participants of Health Behaviour in School-aged Children survey. Complete case analysis limited to 29 countries which participated in both survey waves.

	**Psychological symptom score categories (compared with 0-3 category)**								
	**4-7**			**8-11**			**12-16**		
	**ARR**	**99% CI**	***p***	**ARR**	**99% CI**	***p***	**ARR**	**99% CI**	***p***
**Model not adjusting for individual characteristics[a]**									
Year									
2001-2002	1.00	Ref.	--	1.00	Ref.	--	1.00	Ref.	--
2017-2018	1.09	1.06-1.13	<.001	1.24	1.20-1.29	<.001	1.74	1.65-1.82	<.001
**Model adjusting for sex and age[b]**									
Year									
2001-2002	1.00	Ref.	--	1.00	Ref.	--	1.00	Ref.	--
2017-2018	1.10	1.07-1.14	<.001	1.27	1.22-1.31	<.001	1.79	1.71-1.88	<.001
Sex									
Female	1.00	Ref.	--	1.00	Ref.	--	1.00	Ref.	--
Male	0.74	0.72-0.76	<.001	0.52	0.51-0.54	<.001	0.37	0.36-0.39	<.001
Age, years	1.11	1.11-1.12	<.001	1.18	1.16-1.19	<.001	1.25	1.24-1.27	<.001
**Model adjusting for sex, age, and frequency of social media use[c]**									
Year									
2001-2002	1.00	Ref.	--	1.00	Ref.	--	1.00	Ref.	--
2017-2018	0.97	0.92-1.02	.143	0.94	0.88-1.00	.011	1.06	0.97-1.16	.072
Sex									
Female	1.00	Ref.	--	1.00	Ref.	--	1.00	Ref.	--
Male	0.74	0.72-0.76	<.001	0.52	0.51-0.54	<.001	0.37	0.35-0.39	<.001
Age, years	1.11	1.10-1.12	<.001	1.17	1.16-1.18	<.001	1.24	1.22-1.26	<.001
Frequency of social media use	1.05	1.04-1.07	<.001	1.12	1.10-1.14	<.001	1.19	1.16-1.22	<.001
**Model adjusting for sex, age, and problematic social media use[d]**									
Year									
2001-2002	1.00	Ref.	--	1.00	Ref.	--	1.00	Ref.	--
2017-2018	0.85	0.83-0.87	<.001	0.74	0.73-0.78	<.001	0.77	0.74-0.81	<.001
Sex									
Female	1.00	Ref.	--	1.00	Ref.	--	1.00	Ref.	--
Male	0.75	0.73-0.76	<.001	0.53	0.52-0.54	<.001	0.38	0.36-0.39	<.001
Age, years	1.11	1.10-1.11	<.001	1.16	1.15-1.17	<.001	1.23	1.21-1.24	<.001
Problematic social media use	1.20	1.19-1.21	<.001	1.36	1.35-1.37	<.001	1.50	1.49-1.52	<.001

Abbreviations: ARR stands for adjusted risk ratio, CI for confidence intervals from multinomial logistic regression models in which psychological symptom scores were the outcome of interest and survey wave, the independent variable of interest. Models additionally adjusted for the fixed effect of country (not shown) and for survey weights, stratification, and clustering in classroom.

a. n=310,559.
b. n=308,385.
c. n=284,450.
d. n=285,943.

Online Resource 7: Histograms of variables of frequency of social media use and psychological symptoms as well as their relationship among participants of Health Behaviour in School-aged Children survey, 2017-2018.

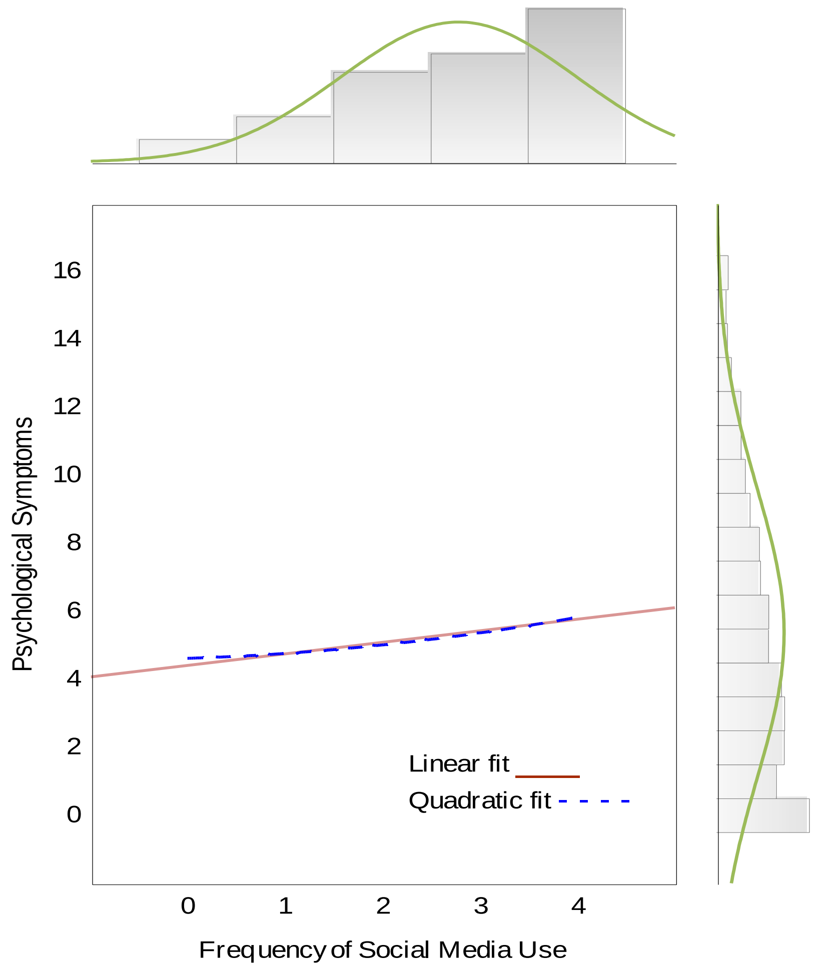

Online Resource 8: Histograms of variables of problematic social media use and psychological symptoms as well as their relationship among participants of Health Behaviour in School-aged Children survey, 2017-2018.

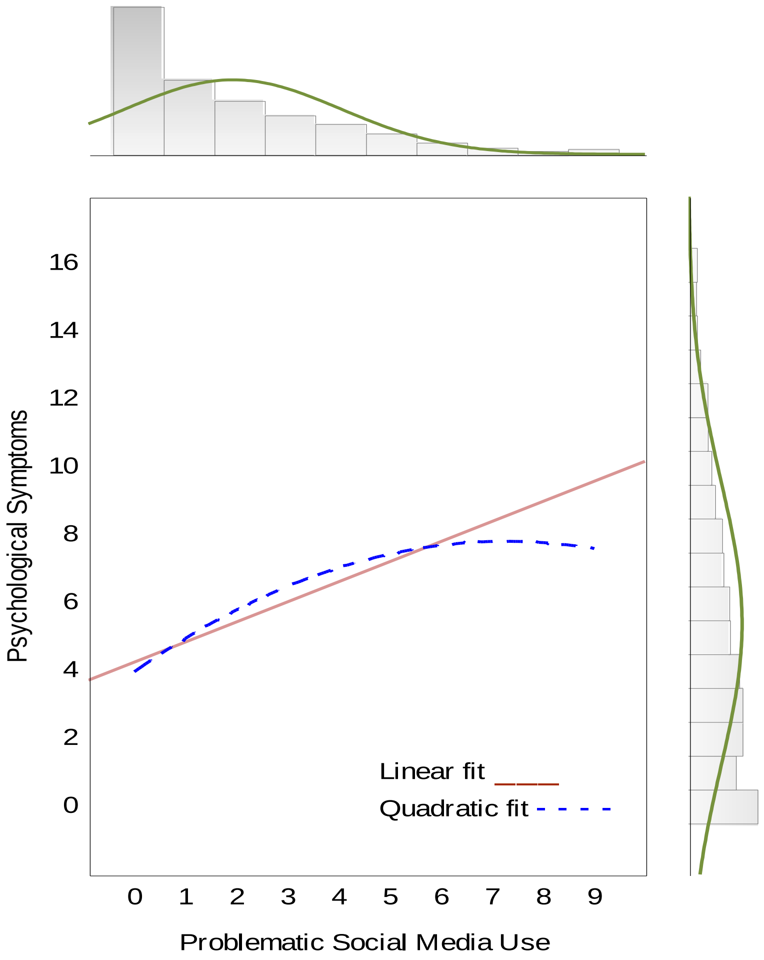

Supplement: Supplementary file 1 — Supplementary file1 (PDF 421 KB) [file 127_2024_2657_MOESM1_ESM.pdf]
